# OpenSim–Umberger-Based Metabolic Power Stratification During the Sit-to-Walk Transition Using Interpretable Ensemble Learning

**DOI:** 10.3390/bioengineering13070774

**Published:** 2026-07-03

**Authors:** Wanli Zang, Jiarong Wu, Jun Wu, Zhengqiu Zhang, Su Wang, Qiuxia Zhang

**Affiliations:** 1 School of Physical Education, Soochow University, Suzhou 215021, China; 2 College of Sports Science and Health, University of Harbin Sport, Harbin 150008, China

**Keywords:** sit-to-walk, metabolic power, OpenSim, Umberger metabolic model, ensemble learning, TOPSIS, SHAP

## Abstract

Quantifying metabolic cost during short transitional movements is challenging because conventional metabolic measurements have limited temporal resolution. This proof-of-concept study examined whether model-derived metabolic cost during the sit-to-walk (STW) transition could be exploratorily stratified using interpretable ensemble learning. Forty-nine healthy adults completed the STW phase of the Timed Up and Go task with synchronized three-dimensional kinematics, ground reaction forces, and eight-channel surface electromyography. Individually scaled OpenSim gait2392 models and the Umberger metabolic model were used to estimate metabolic power from seat-off to the end of the first complete gait cycle. Window-averaged metabolic power was stratified into low-, medium-, and high-cost levels. Window-level biomechanical features were extracted from kinematic, kinetic, and muscle-state time series. Seven classifiers were trained using a subject-level 7:3 train–test split and stratified five-fold cross-validation within the training set, and their probability outputs were integrated through TOPSIS-weighted classifier fusion. SHapley Additive exPlanations were used for class-specific feature attribution. The fused ensemble achieved an AUC of 0.870, F1 score of 0.703, accuracy of 0.705, and specificity of 0.853 on the independent test set. Discrimination was stronger for the low- and high-cost levels than for the medium-cost level. SHAP-based attribution highlighted force-related changes and knee-angle variability and amplitude measures as prediction-relevant biomechanical features. These findings support a model-derived, interpretable workflow for extending STW assessment from task performance to task cost, while indicating the need for further validation in larger and clinical datasets.

## 1. Introduction

The sit-to-walk (STW) task, which involves rising from a seated position and initiating gait, is a common functional activity in daily life [1,2,3]. Unlike steady-state walking, STW is a brief transitional movement that requires coordinated center-of-mass displacement, load transfer, unloading, and propulsion [4,5,6]. Because it places high demands on dynamic stability and lower-limb neuromuscular coordination, STW has been widely used to examine mobility control and rehabilitation-related motor performance [4,7]. Conventional STW assessments, however, mainly describe how fast or how smoothly the task is completed, while offering limited information about the physiological cost required to complete it. Adding task-window-specific metabolic cost to STW assessment could therefore broaden the evaluation from observable performance to the cost of achieving that performance.

In clinical practice, STW is often embedded in the initial phase of standardized functional tests such as the Timed Up and Go (TUG), and its quantification commonly relies on overall TUG outcomes [8,9]. Total completion time is clinically convenient but coarse, and it may not capture fine control strategies within the short transition phase. Instrumented TUG (iTUG) approaches improve assessment resolution by adding kinematic and dynamic information, such as ground reaction forces or center-of-pressure measures, through wearable sensors or force platforms. However, these measures still primarily characterize overt task performance rather than energetic demand [10,11,12]. Individuals with similar completion times or movement trajectories may differ in their internal biomechanical demands and energy requirements [13]. Metabolic cost may therefore complement performance-based measures and provide additional information about how the STW task is executed.

Quantifying energy expenditure during short transitional tasks remains methodologically challenging. Conventional indirect calorimetry based on gas exchange has limited temporal resolution and is affected by physiological response delays, making it difficult to estimate metabolic changes within windows lasting only a few seconds [14]. Simulation-based musculoskeletal modeling offers a potential solution by linking measured movement data to estimated muscle states and metabolic power. Specifically, OpenSim data-tracking simulations can use measured kinematics and kinetics to estimate joint motion, muscle activation, muscle force, and joint moment profiles, which can then be combined with muscle metabolic models such as the Umberger model to derive time-resolved metabolic power estimates [15,16]. Although these estimates are model-derived rather than direct calorimetric measurements, they provide a feasible approach for examining energetic variation during STW.

The biomechanical information relevant to simulation-derived metabolic power during STW is likely to be multidimensional, multisource, and temporally coupled. Window-level feature engineering can summarize kinematic, kinetic, and muscle-state time series into interpretable descriptors, such as change, variability, and amplitude, making these signals more suitable for classification. Machine learning methods are well suited for capturing nonlinear and multivariate patterns in such feature spaces, but prediction alone is insufficient for rehabilitation-oriented interpretation [17,18]. Multi-model ensemble strategies can combine complementary predictions from different classifiers when individual models capture different aspects of biomechanical data. TOPSIS-weighted classifier fusion provides a structured multi-criteria method for ranking and weighting candidate models across multiple performance metrics. SHapley Additive exPlanations (SHAP) can further decompose model predictions into feature-level contributions and help identify biomechanical features most relevant to class discrimination [19,20].

Based on this background, the present study focused on healthy adults and examined a key STW subwindow defined from seat-off to the end of the first complete gait cycle. By integrating experimental measurements with OpenSim–Umberger metabolic modeling, we estimated time-resolved metabolic power within this window and stratified window-averaged metabolic power into low, medium, and high levels in an exploratory manner. We then used a TOPSIS-weighted ensemble learning framework to evaluate whether these simulation-derived metabolic power levels could be distinguished using window-level biomechanical features. Finally, SHAP-based feature attribution was applied to identify prediction-relevant biomechanical indicators contributing to class discrimination. This proof-of-concept study aimed to establish a reproducible workflow integrating model-based metabolic power estimation, exploratory level stratification, ensemble classification, and interpretable feature attribution, thereby providing a healthy-adult methodological baseline for future validation in rehabilitation and clinical populations.

## 2. Materials and Methods

**Figure 1:**
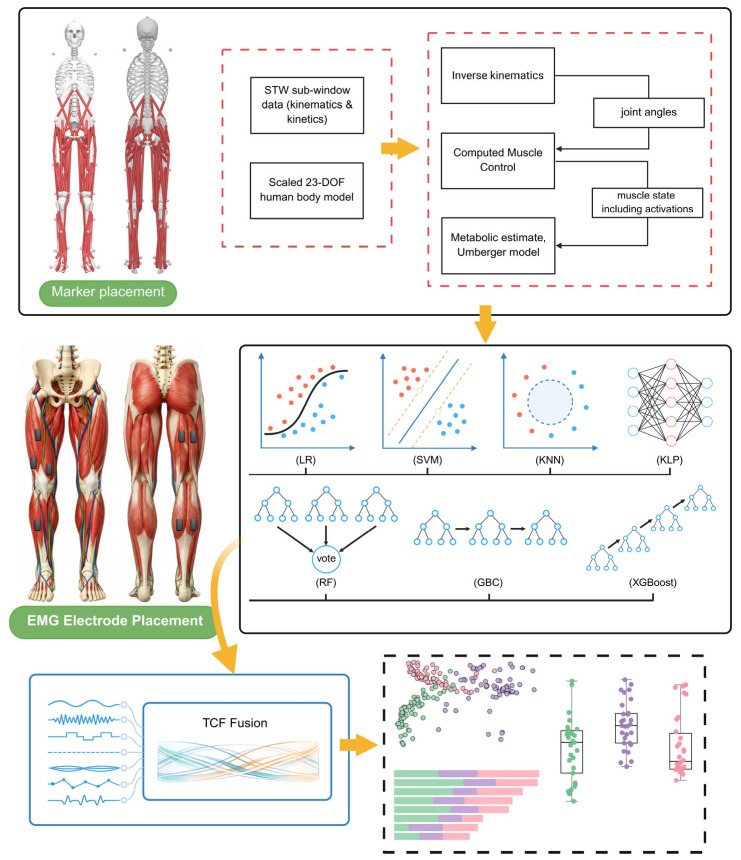
Overview of the analysis workflow.

The overall analysis workflow of this study is illustrated in Figure 1. The STW key transition window was extracted from the TUG task, during which kinematic, kinetic, and surface electromyography (sEMG) data were collected. OpenSim data-tracking simulations combined with the Umberger metabolic model were used to estimate model-derived metabolic cost within the window. Using the simulation-derived cost levels and extracted biomechanical features, multiple classifiers were trained, and low-, medium-, and high-cost levels were explored using a TOPSIS-weighted classifier fusion ensemble model. SHAP was applied for feature attribution to support model interpretation.

### 2.1. Participants and Experimental Procedures

This study was conducted at the Biomechanics Laboratory of Soochow University. The study protocol was reviewed and approved by the Ethics Committee of Soochow University (Approval No: SUDA20251015H11), and all participants provided written informed consent prior to participation. A total of 49 healthy adults were recruited. All participants were able to stand up and walk independently without external assistance and had no apparent motor impairments. Inclusion criteria were: age ≥18 years; normal or corrected-to-normal vision; no history of neurological disorders; no history of musculoskeletal injury or disease; and no severe cardiovascular or respiratory diseases or other chronic conditions that could affect motor control. Exclusion criteria included: neurological or musculoskeletal diseases or injuries that could affect daily mobility; pregnancy; and inability to independently perform standing and walking tasks. Body composition was assessed using an InBody bioelectrical impedance analyzer, and body mass and body mass index (BMI) were recorded.

Three-dimensional kinematic data were collected using a motion capture system comprising 16 infrared cameras (Vicon MX13, Oxford Metrics, Oxford, UK) at a sampling frequency of 100 Hz. Kinetic data were synchronously recorded using four floor-embedded Kistler three-dimensional force plates (model 9281, Kistler Instrumente AG, Winterthur, Switzerland) at 1000 Hz. Reflective markers (14 mm in diameter) were placed on major bony anatomical landmarks of the lower limbs according to the Vicon lower-limb marker set, resulting in a total of 38 single markers (Supplementary Figure S1). sEMG signals were synchronously acquired at 1000 Hz to characterize neuromuscular activity patterns of key lower-limb muscles during the STW task. The motion capture and sEMG systems were synchronized and time-aligned via a unified hardware trigger.

Following previously reported muscle coverage schemes for STW-related studies, sEMG signals were recorded from eight muscles on the dominant lower limb: tibialis anterior (TA), lateral gastrocnemius (GL) and medial gastrocnemius (GM), rectus femoris (RF), vastus lateralis (VL) and vastus medialis (VM), biceps femoris (BF), and semitendinosus (ST). These muscles cover the ankle dorsiflexor and plantarflexor groups, knee extensors, and hamstrings, which are primarily involved in propulsion generation, load transfer, and stability control during STW (Supplementary Figure S1) [21]. sEMG data were collected using YW-wireless bipolar surface electrodes (Zhiyunwei). Electrode placement followed SENIAM recommendations: a single trained examiner identified the target muscle bellies by palpation, cleaned the skin with alcohol swabs, and placed the electrodes along the muscle fiber direction [22]. After electrode placement, participants were asked to perform selective activation of the corresponding muscles to verify signal quality, and electrode positions were adjusted accordingly.

The experimental task was the STW phase at the beginning of the Timed Up-and-Go (TUG) test, defined as the transition from quiet sitting to standing up and initiating forward walking. The initial seated posture was standardized: participants sat on a backless stool adjusted to approximately 90° of knee flexion, with both hands resting on the thighs or knees and both feet placed on the first force plate [3,8]. Upon hearing the start command, participants performed STW and entered walking at a self-selected comfortable speed. To reduce variability related to the leading limb of the first step and to facilitate window alignment, participants were instructed to initiate gait with the left foot after standing. This standardized left-foot initiation was used solely to ensure inter-trial and inter-participant comparability, rather than to define the left limb as the dominant support limb. Upper-limb movements and gait rhythm were not otherwise constrained to preserve natural movement patterns. A representative sequence of the STW task is provided in Supplementary Figure S2. The STW was ultimately divided into four functional phases: P1 (Flexion), P2 (Extension), P3 (Unload), and P4 (Stance). Specifically, P1 corresponded to trunk forward flexion and momentum generation, P2 to trunk and lower-limb extension and lift, P3 to load transfer and swing-limb unloading, and P4 represented the first gait-cycle window after the transition into stance support and propulsion. Each participant completed six successful trials for subsequent analysis. Prior to formal data collection, participants changed into standardized attire and completed approximately 10 min of warm-up and task familiarization. Adequate rest was provided between trials to avoid cumulative fatigue. A successful trial was defined as: (1) no apparent loss of balance or risk of falling throughout the task, and (2) no detachment of key markers or electrodes, and no persistent occlusion or trajectory loss affecting identification of key events.

sEMG signals were processed using a zero-phase second-order Butterworth band-pass filter (20–400 Hz), and kinematic data were filtered using a zero-phase second-order Butterworth low-pass filter with a cutoff frequency of 6 Hz. The STW analysis window was defined from seat-off to the end of the first complete gait cycle. To identify the sitting, sit-to-stand, and stand-to-walk phases and their transition points, event detection was based on bilateral knee joint angle and angular velocity profiles. Periods with low variability in knee angular velocity and small angular excursions were identified as the sitting phase; the sit-to-stand transition was identified when knee joint angles progressively approached extension accompanied by a reduction in angular velocity; and entry into the stand-to-walk phase was determined when knee joint angles and angular velocities exhibited stable periodic oscillations.

In addition to the 49 participants included in the main classification analysis, an independent validation cohort of 19 healthy participants was recruited for oxygen-consumption agreement validation. These participants did not overlap with the main cohort and were not used for feature selection, classifier training, test-set evaluation, or SHAP-based interpretation. In this validation cohort, respiratory metabolic data were recorded synchronously with kinematic data during repeated STW trials using a portable gas-analysis system, MetaMax 3B (CORTEX Biophysik GmbH, Leipzig, Germany).

### 2.2. OpenSim–Umberger-Based Metabolic Cost Modeling

Muscle energy expenditure and metabolic power were estimated using the metabolic probe implemented in OpenSim (Figure 1) [15,23]. The musculoskeletal model gait2392 was adopted [24], which includes three rotational degrees of freedom at the hip joint, one rotational degree of freedom at the knee joint, and two rotational degrees of freedom at both the ankle and subtalar joints. Model scaling was performed by computing scaling factors based on pelvic and foot surface markers, combined with estimated hip joint centers and knee and ankle joint centers, followed by marker adjustments to match individual participant anthropometry [25,26].

Inverse kinematics (IK) was performed using the experimental kinematic data to obtain joint angle trajectories [15,23]. The results of the residual reduction algorithm (RRA) were then used as inputs to computed muscle control (CMC), in which muscle activations were solved via a feedback controller such that forward dynamic simulations simultaneously tracked the experimentally observed kinematics and ground reaction forces [15,27]. Based on the resulting muscle activation and dynamic states, the Umberger metabolic model was applied to estimate the time-varying metabolic power [28,29]. Within the Umberger framework, the metabolic power of an individual muscle is expressed as the sum of the heat production rate and the mechanical power rate, i.e.,

$$\dot{E}=\dot{B}+\sum_{AllMuscles}(\dot{A}+\dot{M}+\dot{S}+\dot{W})$$

where $\dot{B}$ denotes the basal heat rate (W), representing the resting energy expenditure of the muscle; $\dot{A}$ denotes the activation heat rate (W), representing the energy expenditure associated with muscle activation; $\dot{M}$ denotes the maintenance heat rate (W), representing the energy expenditure required to sustain muscle contraction; $\dot{S}$ denotes the shortening heat rate (W), representing the heat produced during muscle shortening; and $\dot{W}$ denotes the mechanical power rate (W), representing the energy expended by the muscle in performing mechanical work (e.g., moving body segments). The Umberger model implemented in OpenSim is a revised version of the original formulation [28], in which metabolic rate is expressed as a function of state variables including muscle activation, muscle fiber length, and shortening velocity.

### 2.3. Candidate Variable Construction and Feature Selection

To avoid data leakage, all feature selection procedures were performed exclusively within the training set [30,31]. First, two categories of strategies were used to evaluate the associations and importance of candidate features with respect to metabolic power levels: (1) filter-based methods, including mutual information and ANOVA F-tests, to characterize nonlinear dependence and linear differences, respectively, between individual features and class labels, and (2) embedded methods, including random forest and XGBoost, from which feature importance was extracted based on ensemble tree models. Feature scores obtained from the four methods were normalized and equally fused to derive a composite importance score. In parallel, a voting mechanism was introduced (selection by at least two methods) to enhance robustness. Ultimately, approximately 20 features that showed stable importance across multiple methods were retained for subsequent three-class modeling (Supplementary Figure S3) [32]. In addition, to further avoid information leakage caused by repeated trials from the same participant being assigned to both the training and test sets, subject-level grouping and separation were strictly applied during all data splitting and cross-validation procedures.

To enhance the model’s ability to capture complex dynamic patterns in movement time-series data, systematic feature engineering was performed based on the original measurements. Although the pointwise temporal order of the raw time series was not explicitly retained, the extracted statistical features effectively captured key temporal dynamics within the gait cycle. Specifically, three categories of window-level summary features were generated: (1) change (Δ) features, which capture dynamic trends over time by computing differences between adjacent time points; (2) standard deviation (SD) features, which quantify variability and fluctuation of the signal within the time window; and (3) range of motion (ROM) features, calculated as the difference between the maximum and minimum values, reflecting the amplitude of variation. These summary features provided richer informational inputs to the fusion model, preserving key temporal dynamics while reducing dimensionality, and facilitated the identification of latent biomechanical patterns embedded in the original data.

### 2.4. Predictive Modeling and TOPSIS-Weighted Fusion

The metabolic power metric exhibited a non-normal distribution based on normality testing; therefore, samples were divided into low, medium, and high groups using tertile thresholds. This grouping strategy preserved gradient information while maintaining relatively balanced class distributions (minimum class proportion > 20%; Supplementary Figure S4). Based on the initial multi-method feature screening, a secondary model-based selection step was applied within the training set to further reduce redundancy and the risk of overfitting. This step resulted in eight key features retained as input variables: Latitudinal Force Left Delta, Medial Force Right Delta, Latitudinal Force Right Delta, Medial Force Left Delta, Standard Deviation of Right Knee Angle, Range of Right Knee Angle, Standard Deviation of Left Latitudinal Force, and Standard Deviation of Left Medial Force. Participants, rather than individual trials, were randomly assigned to the training and independent test sets at an approximately 7:3 ratio, while approximately preserving class proportions across the three groups. Specifically, the training set consisted of 34 participants (Low: 12, Medium: 11, High: 11), and the test set consisted of 15 participants (Low: 5, Medium: 5, High: 5). The confusion matrices in the Results Section report window-level counts, not participant-level counts. Participant-level splitting was used only to prevent data leakage.

Seven machine learning classifiers were constructed and compared: random forest (RF), support vector machine (SVM), XGBoost, logistic regression (LR), gradient boosting classifier (GBC), k-nearest neighbors (KNN), and multilayer perceptron (MLP). Z-score standardization was applied to SVM, KNN, LR, and MLP, whereas tree-based models (RF, XGBoost, and GBC) were trained in the original feature space. All models were trained using stratified group five-fold cross-validation within the training set, with the macro-averaged F1 score as the optimization objective. Optimal hyperparameter combinations were determined via grid search (GridSearchCV). Models were then retrained on the full training set using the optimal parameters, and generalization performance was evaluated on the independent test set. Model performance was comprehensively assessed using AUC, F1, precision (PRE), accuracy (ACC), sensitivity (SEN), specificity (SPE), as well as positive predictive value (PPV) and negative predictive value (NPV). Confusion matrices (rows representing true classes and columns representing predicted classes) were constructed to analyze misclassification patterns among the three metabolic power levels.

Given the complementarity of individual models across different performance metrics, a TOPSIS-based weighted fusion model, termed TCF, was further constructed to integrate the output probabilities of the seven submodels (M = 7). First, TOPSIS composite scores were computed for each submodel based on their performance across six evaluation metrics. These composite scores were then normalized to obtain submodel weights. Finally, the class probability outputs of the submodels were combined using a weighted summation based on the derived weights to yield the TCF predicted probabilities. For each sample, the class with the highest predicted probability was taken as the final classification result of the TCF model.

AUCi, F1-scorei, ACCi, PREi, SENi, and SPEi (i = 1, 2, …, M) were used to denote the performance of the i-th submodel on the six evaluation metrics. All of these metrics are benefit-type criteria (i.e., larger values indicate better performance); therefore, no preprocessing such as minimization or sign reversal was required. We constructed the evaluation matrix:

$$X\;=\;(x_{\mathrm{ij}}{)}_{M\times 6}$$

where $x_{i1}\;=\;{\mathrm{AUC}}_{i},\;x_{i2}\;=\;F1-{\mathrm{score}}_{i},\;x_{i3}\;=\;{\mathrm{ACC}}_{i},\;x_{i4}\;=\;{\mathrm{PRE}}_{i},\;x_{i5}\;=\;{\mathrm{SEN}}_{i},\;x_{i6}\;=\;{\mathrm{SPE}}_{i}.$ To eliminate the influence of different metric scales, column-wise vector normalization was applied to matrix X. The normalized value is denoted as $r_{\mathrm{ij}}$,which was calculated as:

$$r_{\mathrm{ij}}\;=\frac{x_{\mathrm{ij}}}{\sqrt{\sum_{k=1}^{M}x_{\mathrm{kj}}^{2}}},\;i\;=\;1,\ldots ,\;M;\;j\;=\;1,\ldots ,6$$

On this basis, equal weights were assigned to each metric, yielding the weight vector:

$$w\;=\left[\begin{array}{c} \frac{1}{6},\frac{1}{6},\frac{1}{6},\frac{1}{6},\frac{1}{6},\frac{1}{6} \end{array}\right]$$

The weighted normalized matrix was then obtained as:

$$v_{\mathrm{ij}}\;=\;w_{j}r_{\mathrm{ij}},\;j\;=\;1,\ldots ,6$$

For each metric j, the maximum and minimum values across all submodels were identified to form the positive ideal solution (positive ideal solution, PIS) and the negative ideal solution (negative ideal solution, NIS), respectively:

$$v_{j}^{+}\;=\underset{1\;\leq \;i\;\leq \;M}{\max}v_{\mathrm{ij}}$$

$$v_{j}^{-}=\underset{1\;\leq \;i\;\leq \;M}{\min}v_{\mathrm{ij}}$$

Accordingly:

$$\mathrm{PIS}\;=\;\left(v_{1}^{+},\;v_{2}^{+},{\;\mathrm{PRE}}_{\max},{\;\mathrm{ACC}}_{\max},{\;\mathrm{SEN}}_{\max},{\;\mathrm{SPE}}_{\max}\right)$$

$$\mathrm{NIS}=\left(v_{1}^{-},{\;v}_{2}^{-},{\;\mathrm{PRE}}_{\min},{\;\mathrm{ACC}}_{\min},{\;\mathrm{SEN}}_{\min},\;{\mathrm{SPE}}_{\min}\right)$$

The TOPSIS method was then used to compute the composite score for each model. The Euclidean distances between the i-th submodel and the PIS and NIS were calculated as:

$$d_{i}^{+}\;=\;\sqrt{\sum_{j\;=\;1}^{6}(v_{\mathrm{ij}\;}-{\;v}_{j}^{+}{)}^{2}}$$

$$d_{i}^{-}=\sqrt{\sum_{j=1}^{6}(v_{\mathrm{ij}\;}-v_{j}^{-}{)}^{2}}$$

The TOPSIS composite score was defined as:

$${\mathrm{topsis}\text{\_}\mathrm{score}}_{i}=\;c_{i}\;=\frac{d_{i}^{-}}{d_{i}^{+}\;+\;d_{i}^{-}},\;i\;=\;1,\;2,\ldots ,\;M.$$

For model fusion, the composite scores were normalized to obtain the weight of each submodel, denoted as $\alpha_{i}$:

$$\alpha_{i}\;=\;\frac{c_{i}}{\sum_{k\;=\;1}^{M}c_{k}},\;i\;=\;1,\;2,\ldots ,\;M$$

Accordingly, $\;\alpha_{i}$ ≥ 0 and $\sum_{i\;=\;1}^{M}\alpha_{i}\;=\;1$.

In the model fusion stage, let $p_{\mathrm{ijc}}$ denote the predicted probability produced by the i-th submodel that the j-th participant belongs to class c (c = 1, 2, 3). The fused probability generated by the TCF model that the j-th participant belongs to class c was computed as:

$${\hat{p}}_{\mathrm{jc}}=\sum_{i\;=\;1}^{M}\alpha_{i}p_{\mathrm{ijc}},\;j\;=\;1,2,\ldots ,N;c\;=\;1,2,3$$

Finally, for each participant, the class corresponding to the maximum component of the fused probability vector $\left({\hat{p}}_{j1},{\hat{p}}_{j2},{\hat{p}}_{j3}\right)$ was taken as the TCF classification result [33].

### 2.5. Model Interpretation

SHAP values were used to analyze the contribution of each feature to the model output. SHAP values assign an attribution value to each feature, quantitatively measuring its impact on the prediction. Specifically, the model prediction f(x) can be expressed as:

$$g(z^{\prime })\;=\;\vartheta_{0\;}+\;\sum_{i\;=\;1}^{M}\vartheta_{i}z_{i}^{\prime }$$

where $g(z^{\prime })$ denotes the explanation model, $\vartheta_{i}$ represents the Shapley value of feature i, and $z_{i}^{\prime }$ is a binary variable indicating whether feature i is present in the model. For the three-class classification problem, SHAP values were computed separately for each class. These SHAP values enabled quantitative assessment of the contribution of each feature to predictions for different classes and facilitated comparison of feature contribution patterns across classes.

### 2.6. Statistical Analysis

The Kolmogorov–Smirnov test was used to assess whether each variable followed a normal distribution [34]. When data did not conform to normality, differences between categories were evaluated using the Mann–Whitney U test, with Bonferroni correction applied to control for false-positive errors introduced by multiple comparisons [35]. Model-performance uncertainty was assessed using bootstrap resampling of the independent test set. For each model, AUC, F1 score, accuracy, precision, sensitivity, specificity, PPV, and NPV were recalculated across repeated bootstrap samples and summarized as mean ± SD. Pairwise differences in macro-averaged AUC between the TCF model and individual base models were assessed using Wilcoxon signed-rank tests, with Holm correction applied for multiple comparisons. Paired classification outputs between the TCF model and the strongest individual base model were further compared using McNemar’s test. A two-sided *p* value less than 0.05 was considered statistically significant.

## 3. Results

### 3.1. Participant Characteristics and Metabolic Power Stratification

A total of 49 healthy participants were included in this study, all of whom completed the STW phase of the TUG test. The mean metabolic power within the STW key window was used as the stratification criterion, and trials were divided into low, medium, and high levels using tertile thresholds to preserve gradient information of metabolic load while achieving a relatively balanced class distribution. Demographic and anthropometric characteristics of the participants, as well as the grouping information, are presented in Table 1.

### 3.2. Validation of Data-Tracking Simulation Results

To evaluate the data-tracking simulation and OpenSim–Umberger metabolic-estimation workflow, the independent validation cohort described above was used. The main classification analysis was still based on the 49 participants, whereas the additional cohort was used only for validation. To assess the quality of the data-tracking simulation, experimentally measured joint-angle trajectories within the STW analysis window were compared with the corresponding OpenSim simulation outputs. The experimental and simulated trajectories showed good agreement in the main joint-angle patterns, with R^2^ values ranging from 0.904 to 0.976 (Supplementary Figure S5). In addition, to examine the consistency of neuromuscular timing, preprocessed sEMG signals were time-aligned and compared with model-estimated muscle activations, and similarity indices were calculated (Supplementary Figure S6). For metabolic validation, the model-estimated metabolic rate showed overall agreement with oxygen-consumption-derived metabolic measures (R^2^ = 0.804; Supplementary Figure S7). These results provided supportive evidence for the feasibility of using the OpenSim–Umberger workflow to estimate metabolic energy expenditure during individual movement tasks.

### 3.3. Baseline Kinematic, Kinetic, and sEMG Features Across Metabolic-Cost Groups

**Figure 2:**
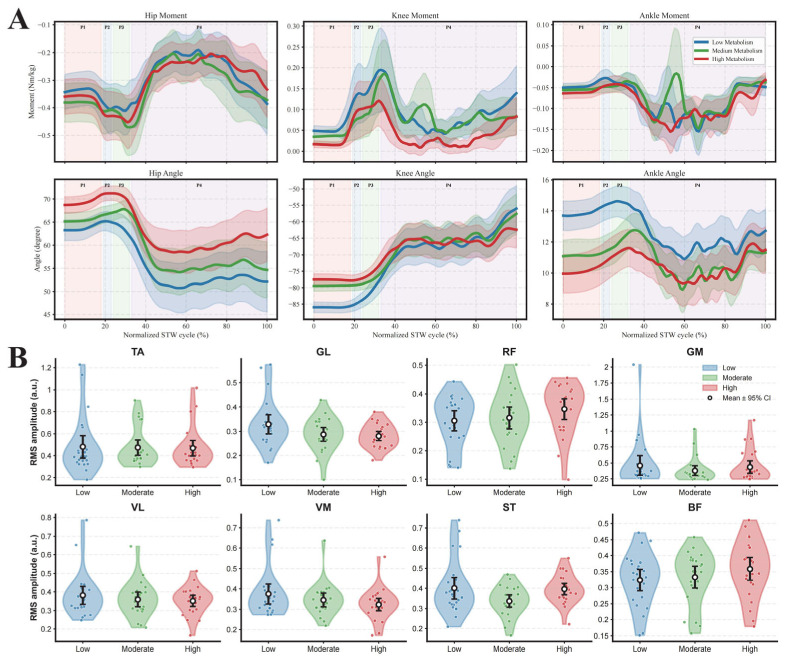
Kinematic, kinetic, and sEMG features during the STW task across metabolic-cost levels. (**A**) Hip, knee, and ankle joint moment and angle trajectories over the normalized STW cycle. Curves represent group means, and shaded areas indicate 95% confidence intervals. Background colors indicate P1 (flexion), P2 (extension), P3 (unloading), and P4 (stance). Joint moments were normalized to body weight. (**B**) Between-group distributions of sEMG RMS amplitudes for eight major muscles of the right lower limb. Violin plots indicate data distributions, scattered points indicate participant-level values, and white circles with error bars indicate means ±95% confidence intervals.

Figure 2 shows the hip, knee, and ankle joint angle and moment profiles over the normalized STW cycle (0–100%), as well as the sEMG RMS amplitude distributions of the main muscles of the right lower limb. Overall, the kinematic, kinetic, and sEMG variables exhibited clear phase-dependent patterns, with between-group differences observed in specific phases. These descriptive patterns provided baseline information for subsequent feature construction and model interpretation.

### 3.4. Three-Class Model Performance and Ensemble Model Results

The test-set performance of the individual base models and the TCF is summarized in Table 2. The TCF model achieved an AUC of 0.870, an F1 score of 0.703, an accuracy of 0.705, a precision of 0.704, a sensitivity of 0.705, and a specificity of 0.853. Among the individual models, KNN yielded the highest AUC (0.873), but its F1 score (0.677) and accuracy (0.682) were lower than those of the TCF model. SVM showed the highest F1 score (0.690) and accuracy (0.693) among the individual models, but these values also remained lower than those of the TCF model. Pairwise comparisons using Holm-corrected Wilcoxon signed-rank tests showed significant differences in macro-averaged AUC between the TCF model and each individual base model (all adjusted *p* < 0.001). McNemar’s test further indicated a significant difference in paired classification outputs between the TCF model and SVM, the strongest individual model based on F1 score and accuracy (*p* < 0.001). Overall, the TCF model achieved the most balanced performance across F1 score, accuracy, precision, sensitivity, specificity, PPV, and NPV. Hyperparameter settings for all submodels are provided in Supplementary Table S1.

**Figure 3:**
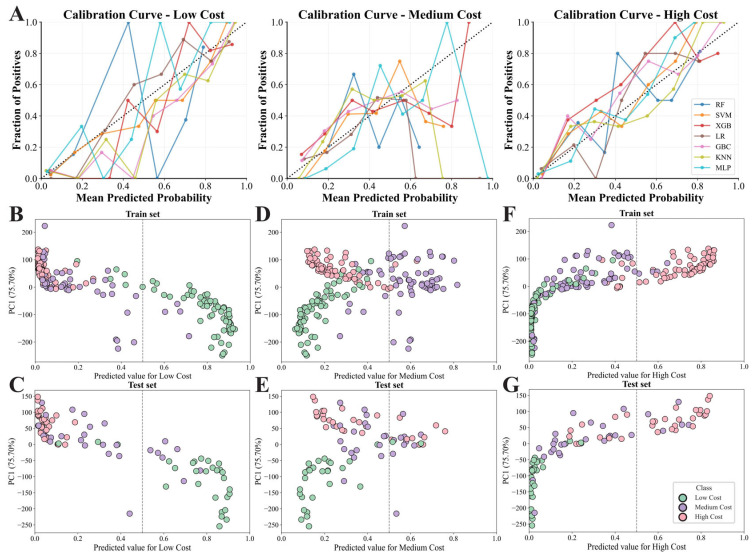
Model calibration and predicted probability distributions. (**A**) Calibration curves for the three metabolic power classes across all models. (**B**–**G**) Distributions of predicted class probabilities in the training and test sets.

Calibration curves for the three metabolic power classes across all models are presented in Figure 3A. Figure 3B–G illustrate the distributions of predicted probabilities in the training and test sets, along with sample distributions visualized using principal component analysis (PCA). The first principal component (PC1) explained approximately 73–75% of the variance in both the training and test sets. In the low-dimensional space, low- and high-cost samples exhibited relatively clearer separation. In contrast, medium-cost samples showed a more dispersed distribution, with greater overlap with other classes in the test set. Moreover, their maximum predicted class probabilities were generally lower or closer to the second-highest class probabilities, indicating relatively greater difficulty in discriminating this class.

### 3.5. Class-Specific Discrimination Performance

**Figure 4:**
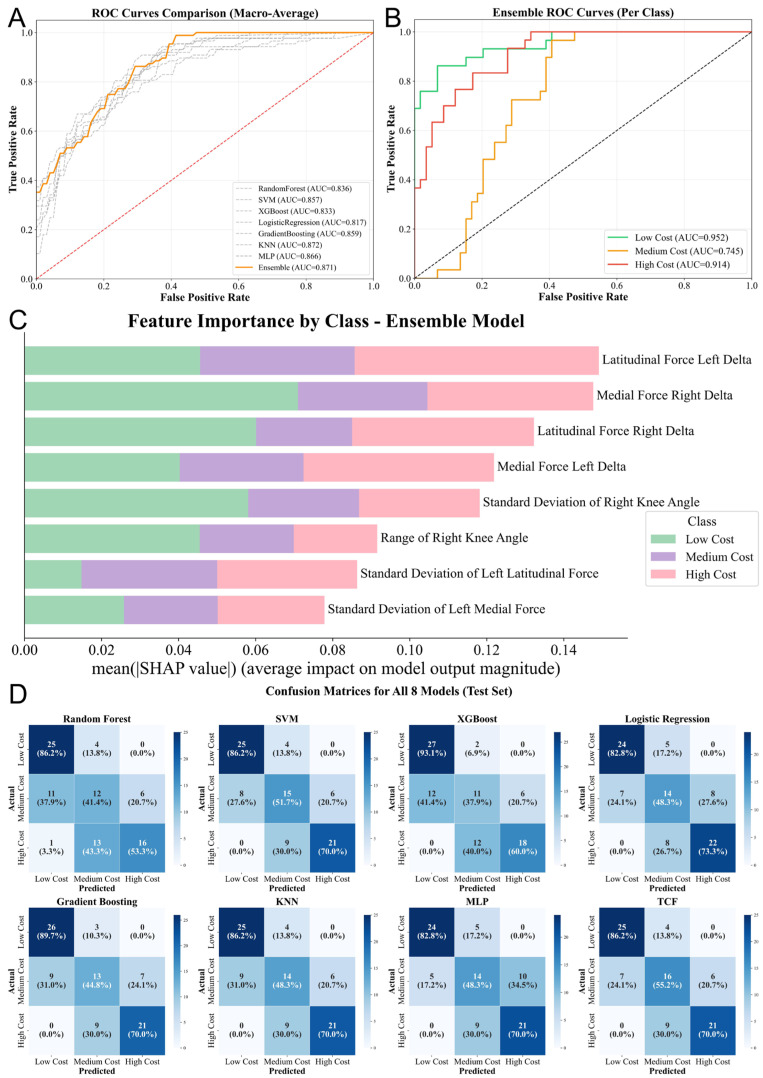
Performance, feature importance, and classification results of the ensemble model. (**A**) Comparison of macro-averaged ROC curves between the ensemble model and individual base models on the test set. (**B**) One-vs-rest ROC curves of the ensemble model for low-, medium-, and high-cost classes with corresponding AUCs. (**C**) SHAP feature importance of the ensemble model, showing mean absolute SHAP values for the top eight features in metabolic-cost classification. Feature labels refer to force-related metrics of the vastus lateralis (Lat) and vastus medialis (Med) muscles on the left (L) and right (R) sides; Delta denotes within-window change, Std denotes standard deviation, and Range denotes knee angle range of motion. (**D**) Confusion matrices of all eight models on the test set. All values represent window-level counts (not participants or trials).

The comparison of macro-averaged ROC curves between the TCF ensemble model and the individual base models is shown in Figure 4A, with the TCF achieving a macro-averaged AUC of 0.871. Further one-vs-rest (OvR) ROC analyses by class (Figure 4B) demonstrated class-dependent discrimination performance of the TCF model: discrimination was strong for the low-cost (AUC = 0.952) and high-cost (AUC = 0.914) levels, whereas discrimination for the medium-cost level was relatively weaker (AUC = 0.745).

### 3.6. Feature Contributions and Interpretability

SHAP importance results indicated that model-estimated muscle-force change features and knee-angle descriptors made prominent contributions to model discrimination (Figure 4C). Among the top eight most important features, four were within-window quadriceps-related muscle-force change features, including Latitudinal Force Left Delta, Medial Force Right Delta, Latitudinal Force Right Delta, and Medial Force Left Delta. Two features described right knee angle variability and amplitude, namely Standard Deviation of Right Knee Angle and Range of Right Knee Angle. The remaining two features were variability descriptors of left quadriceps-related muscle-force features, including Standard Deviation of Left Latitudinal Force and Standard Deviation of Left Medial Force. At the class level, these features showed different contribution patterns across the low-, medium-, and high-cost class outputs. Overall, the global SHAP ranking suggests that the ensemble model used quadriceps-related muscle-force changes and knee-angle variability or amplitude measures as prediction-relevant biomechanical features for metabolic-cost classification during the STW transition.

### 3.7. Classification Error Patterns

Confusion matrices for all eight models on the test set are presented in Figure 4D. For the TCF model, correct classification rates were 25/29 (86.2%) for the low-cost class, 16/29 (55.2%) for the medium-cost class, and 21/30 (70.0%) for the high-cost class. Among medium-cost samples, 7/29 (24.1%) were classified as low cost and 6/29 (20.7%) were classified as high cost. Among high-cost samples, 9/30 (30.0%) were classified as medium cost. No direct misclassification occurred between the low- and high-cost classes, with no low-cost samples classified as high cost and no high-cost samples classified as low cost. Overall, classification errors were concentrated around the medium-cost class.

### 3.8. Class-Specific SHAP Value Distributions

**Figure 5:**
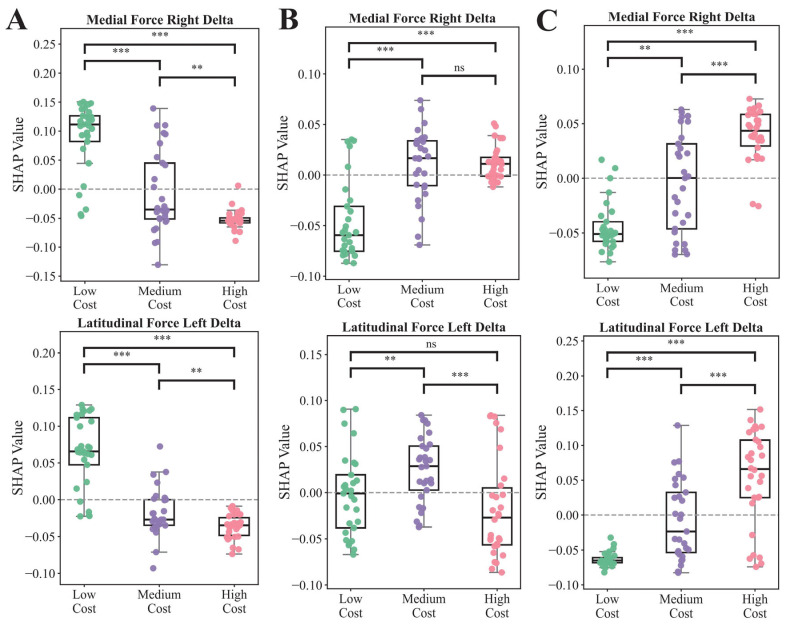
Class-specific SHAP value distributions for two key features, Latitudinal Force Left Delta and Medial Force Right Delta, across metabolic-cost levels. Panel (**A**) shows SHAP values for the low-cost class output; Panel (**B**) shows SHAP values for the medium-cost class output; Panel (**C**) shows SHAP values for the high-cost class output. Within each panel, samples are grouped according to their true metabolic-cost level. Pairwise comparisons were performed using Mann–Whitney U tests with Bonferroni correction. Significance levels were based on Bonferroni-adjusted *p* values and are indicated as ** *p* < 0.01, and *** *p* < 0.001; ns indicates no statistical significance.

Figure 5 presents the class-specific SHAP value distributions of Latitudinal Force Left Delta and Medial Force Right Delta across the true low-, medium-, and high-cost groups. In the low-cost class output (Figure 5A), both features showed positive SHAP values mainly in the low-cost group and negative SHAP values mainly in the high-cost group, with all pairwise comparisons reaching significance. In the medium-cost class output (Figure 5B), the distributions showed greater overlap across groups. Medial Force Right Delta differed significantly between the low- and medium-cost groups and between the low- and high-cost groups, but not between the medium- and high-cost groups. Latitudinal Force Left Delta differed significantly between the low- and medium-cost groups and between the medium- and high-cost groups, but not between the low- and high-cost groups. In the high-cost class output (Figure 5C), both features showed higher SHAP values mainly in the high-cost group, and all pairwise comparisons were significant. Overall, non-significant pairwise comparisons were observed only in the medium-cost class output.

## 4. Discussion

### 4.1. Main Findings

This proof-of-concept study developed and evaluated an integrated analytical workflow for estimating and stratifying model-derived metabolic cost during the STW transition. Focusing on the subwindow from seat-off to the end of the first complete gait cycle, OpenSim data-tracking simulations combined with the Umberger metabolic model were used to estimate time-resolved metabolic power, E(t), and window-averaged values were stratified into low-, medium-, and high-cost levels in an exploratory manner. The TOPSIS-weighted classifier fusion model showed stable overall discrimination on the independent test set, with stronger performance for the low- and high-cost classes than for the medium-cost class. SHAP-based feature attribution further indicated that window-level biomechanical descriptors, particularly force-related changes and knee-angle variability or amplitude measures, contributed to class discrimination. However, the weaker discrimination and greater overlap observed for the medium-cost class suggest that the intermediate range may represent a transitional zone along a continuous metabolic-cost spectrum rather than a clearly separable category.

### 4.2. From STW Performance to Model-Derived Metabolic Cost

Previous quantification of STW/TUG performance has largely relied on total completion time, spatiotemporal parameters, and joint kinematic or kinetic measures to characterize motor control [3,8,36]. Although these measures are clinically useful, total time alone may have limited sensitivity to subtle control deficits during short transitional movements [12,37,38]. Segmenting the TUG task and extracting subtask-specific metrics for sit-to-stand, straight walking, turning, and stand-to-sit phases can provide a more detailed characterization of mobility capacity and control features [39]. Building on this perspective, the present study focused on a phase-aligned STW window and used a model-derived metabolic-cost measure to complement conventional performance-based assessment.

The findings suggest that energetic demand within the STW transition window may provide information that is not fully captured by external performance metrics alone. This interpretation is consistent with gait energetics research showing that metabolic energy expenditure can vary with control strategies even when global gait appearance or speed changes only modestly [40]. Therefore, incorporating model-derived metabolic cost into STW assessment may extend evaluation from task completion to the energetic demand required to achieve that completion. In this study, the window-averaged cost measure provided a model-derived summary of STW-related metabolic demand and supported exploratory stratification across the transition window.

### 4.3. Ensemble Fusion for Multisource Biomechanical Classification

In the model comparison, the TOPSIS-weighted classifier fusion model showed stable overall discrimination on the independent test set, with relatively stronger classification of the low- and high-cost levels than of the medium-cost level. This finding supports the potential value of ensemble strategies for biomechanical data that include multiple feature sources and nonlinear structures [41,42]. Human movement data are typically high-dimensional and heterogeneous, and different classifiers may capture different aspects of the feature space [17]. For example, margin-based, tree-based, and instance-based models may differ in their sensitivity to decision boundaries, nonlinear interactions, and local data structure. Similar observations have been reported in biosignal classification, where classifier performance can depend on the type of extracted features [43].

Within this context, TOPSIS-weighted fusion provides a structured way to integrate model outputs across multiple performance metrics, thereby reducing reliance on a single classifier and its specific assumptions [33]. However, the advantage of the ensemble model should be interpreted cautiously. The current analysis was exploratory, and the magnitude of improvement over individual models requires confirmation in larger and external datasets.

### 4.4. Prediction-Relevant Biomechanical Feature Attribution

SHAP-based feature attribution indicated that several window-level biomechanical descriptors contributed to class discrimination. These features mainly included force-related change measures and knee-angle variability or amplitude measures. This pattern suggests that the model relied not only on static mean values or isolated peaks but also on descriptors reflecting changes and fluctuations within the STW transition window.

Force-related change features may reflect the rapid redistribution of lower-limb loading and support demands during the transition from rising to gait initiation. Compared with steady-state walking, STW requires load transfer, postural stabilization, and preparation for the first step within a short time window [44]. Previous work has also suggested that stability control during locomotion can be associated with increased metabolic demand [45]. In the present study, these features should therefore be interpreted as prediction-relevant biomechanical indicators associated with classification, rather than as causal determinants of metabolic cost.

Knee-angle variability and amplitude measures may provide additional information about movement regulation during the transition. Prior gait energetics research suggests that greater movement variability may accompany higher energetic demand under some conditions [46]. Consistent with the present results, class-specific SHAP distributions showed clearer feature contribution patterns for the low- and high-cost outputs, whereas the medium-cost output showed greater overlap. This supports the interpretation that the intermediate cost range may be less clearly separable than the two extreme ranges.

### 4.5. Limitations and Future Directions

Several limitations should be considered when interpreting the present findings. First, although subject-level separation was used to reduce information leakage between training and testing procedures, the number of independent participants remained limited. The inclusion of repeated trials increased the number of observations for model development, but it did not fully overcome the limited size of the independent participant sample. Therefore, the reported performance and feature-attribution patterns should be confirmed in larger datasets with independent external validation.

Second, the study population consisted of healthy adults. This design was appropriate for establishing an initial methodological baseline, but it limits direct generalization to older adults or patients with neurological, musculoskeletal, or balance-related impairments. Future studies should test whether the proposed workflow remains stable in clinical populations, in whom compensatory strategies, impaired force generation, and altered neuromuscular control may change both metabolic demand and classification-relevant biomechanical features.

Third, the metabolic-cost labels were derived from OpenSim–Umberger simulations rather than direct calorimetric measurements. Although the supplementary oxygen-consumption validation provided supportive evidence for the consistency between model-derived estimates and oxygen-consumption-derived metabolic measures, these quantities are not identical. The validation subset was also limited in size. Thus, the present findings support the plausibility of the model-derived estimates but do not replace larger-scale validation using independent physiological measurements.

Fourth, the low-, medium-, and high-cost levels were defined using tertiles of window-averaged metabolic cost. This strategy provided balanced groups for exploratory classification, but it discretized an underlying continuous variable. The weaker performance and greater overlap observed for the medium-cost class suggest that the intermediate range may represent a transition zone along a continuous metabolic-cost spectrum rather than a clearly separable category. Future work should therefore compare tertile-based classification with continuous regression, ordinal regression, data-driven clustering, and low-versus-high sensitivity analyses.

Finally, SHAP-based feature attribution should be interpreted as an explanation of model predictions rather than as evidence of causal effects on metabolic cost. The identified force-related and knee-angle features may help characterize classification-relevant biomechanical patterns, but causal interpretation would require experimental manipulation, longitudinal validation, or mechanistic intervention studies. In addition, the present analysis used window-level summary features such as change, variability, and amplitude. Future studies may further incorporate time-series models or deep learning architectures when larger datasets become available, while maintaining subject-level validation and transparent reporting of preprocessing, feature selection, and model evaluation procedures.

## 5. Conclusions

In this proof-of-concept study of 49 healthy participants performing the TUG–STW task, we focused on the transition window from seat-off to the end of the first complete gait cycle and estimated model-derived metabolic cost using OpenSim data-tracking simulations combined with the Umberger metabolic model. Window-averaged metabolic cost was stratified into low-, medium-, and high-cost levels in an exploratory manner. Using window-level statistical features derived from kinematics, ground reaction forces, and sEMG, the TOPSIS-weighted classifier fusion framework showed stable overall discrimination on the independent test set, with stronger performance for the low- and high-cost levels than for the medium-cost level. SHAP-based feature attribution highlighted force-related changes and knee-angle variability or amplitude measures as prediction-relevant biomechanical features contributing to class discrimination. These findings suggest that simulation-derived metabolic-cost estimation, ensemble classification, and interpretable feature attribution may provide a methodological basis for extending STW assessment from task performance to task cost. Future studies should validate this workflow in larger independent and clinical datasets and compare tertile-based classification with ordinal, continuous, or low-versus-high modeling strategies, particularly for characterizing the intermediate cost range.

## Figures and Tables

**Table 1:** Demographic and anthropometric characteristics of the participants.

Variables	Low Cost	Medium Cost	High Cost
Age (Years)	34.97 ± 15.23	40.14 ± 18.4	43.55 ± 18.98
Weight (kg)	72.95 ± 12.8	65.38 ± 14.8	56.98 ± 9.69
Height (m)	1.7 ± 0.06	1.63 ± 0.1	1.58 ± 0.07
BMI (kg/m^2^)	25.22 ± 4.13	24.35 ± 3.76	22.68 ± 3.12
Metabolic Power Index (Medium Cost = 100)	99.76 ± 4.16	100.00 ± 3.87	103.62 ± 4.15

Note: Values are presented as mean ± SD.

**Table 2:** Model Performance on Test Set.

Model	AUC	F1-Score	ACC	PRE	SEN	SPE	PPV	NPV
RandomForest	0.8356 ± 0.0301	0.5956 ± 0.0182	0.6023 ± 0.0493	0.6056 ± 0.0164	0.6031 ± 0.0480	0.8017 ± 0.0279	0.6056 ± 0.0164	0.8071 ± 0.0164
SVM	0.8566 ± 0.0302	0.6899 ± 0.0485	0.6932 ± 0.0498	0.6904 ± 0.0488	0.6931 ± 0.0478	0.8469 ± 0.0256	0.6904 ± 0.0488	0.8488 ± 0.0256
XGBoost	0.8326 ± 0.0323	0.6227 ± 0.0500	0.6364 ± 0.0527	0.6274 ± 0.0522	0.6368 ± 0.0462	0.8186 ± 0.0268	0.6274 ± 0.0522	0.8287 ± 0.0266
LogisticRegression	0.8174 ± 0.0326	0.6778 ± 0.0483	0.6818 ± 0.0499	0.6753 ± 0.0496	0.6812 ± 0.0474	0.8410 ± 0.0258	0.6753 ± 0.0496	0.8428 ± 0.0258
GradientBoosting	0.8592 ± 0.0295	0.6727 ± 0.0494	0.6818 ± 0.0509	0.6710 ± 0.0514	0.6816 ± 0.0469	0.8411 ± 0.0261	0.6710 ± 0.0514	0.8465 ± 0.0258
KNN	0.8725 ± 0.0292	0.6768 ± 0.0492	0.6818 ± 0.0505	0.6772 ± 0.0502	0.6816 ± 0.0481	0.8412 ± 0.0259	0.6772 ± 0.0502	0.8442 ± 0.0259
MLP	0.8658 ± 0.0301	0.6691 ± 0.0492	0.6705 ± 0.0518	0.6683 ± 0.0500	0.6701 ± 0.0494	0.8352 ± 0.0274	0.6683 ± 0.0500	0.8358 ± 0.0275
Ensemble	0.8705 ± 0.0372	0.7027 ± 0.0182	0.7045 ± 0.0493	0.7036 ± 0.0164	0.7046 ± 0.0450	0.8525 ± 0.0214	0.7036 ± 0.0164	0.8536 ± 0.0164

Note: AUC denotes the area under the receiver operating characteristic curve; F1-score represents the harmonic mean of precision and recall; ACC indicates accuracy; PRE refers to precision; SEN denotes sensitivity (recall); SPE represents specificity; PPV indicates positive predictive value; and NPV denotes negative predictive value.

## Data Availability

The data supporting the findings of this study are not publicly available due to participant privacy and ethical restrictions. The data may be made available from the corresponding authors upon reasonable request and with permission from the relevant ethics committee or institution.

## Supplementary Materials

The following supporting information can be downloaded at: https://www.mdpi.com/article/10.3390/bioengineering13070774/s1, Figure S1: Reflective marker configuration and surface electromyography (sEMG) electrode placement; Figure S2: Representative sequence of the sit-to-walk task; Figure S3: Feature selection workflow and results; Figure S4: Distribution of window-averaged metabolic power and tertile-based stratification into low, medium, and high groups, showing preserved gradient information and balanced class proportions (minimum class proportion > 20%); Figure S5: Comparison of Vicon-based and OpenSim-derived lower-limb joint angles during the cycle; Figure S6: Group-averaged comparison of EMG envelopes and OpenSim-simulated activations of representative lower-limb muscles during a complete gait cycle; Figure S7. Comparison of OpenSim-Umberger model-estimated metabolic rate and indirect calorimetry-measured oxygen consumption across five repeated STW trials.Trial labels (Trial 1–5) correspond to the original chronological order of the repeated STW trials performed during the experiment and were not reordered for analysis. Table S1: Best Hyperparameters for Each Model.

## Abbreviations

The following abbreviations are used in this manuscript:

iTUG	Instrumented Timed Up and Go
sEMG	Surface Electromyography
OpenSim	Musculoskeletal Model
SHAP	Shapley Additive Explanations
RF	Random Forest
SVM	Support Vector Machine
XGBoost	XGBoost
LR	Logistic Regression
GBC	Gradient Boosting Classifier
KNN	K-Nearest Neighbors
MLP	Multilayer Perceptron
TOPSIS-based TCF	Top Ranked and Ranked Weighted Classification Fusion
ROM	Range of Motion
SD	Standard Deviation
Δ	Change (difference between adjacent time points)
