# Supplementary material for: OpenSim–Umberger-Based Metabolic Power Stratification During the Sit-to-Walk Transition Using Interpretable Ensemble Learning
Source: Bioengineering (Basel). 2026 Jul 3;13(7):774. doi: 10.3390/bioengineering13070774 (PMC13405875; doi:10.3390/bioengineering13070774)
Supplement: Supplementary file 1 [file bioengineering-13-00774-s001.zip › bioengineering-4356556-supplementary.pdf]

Supplementary

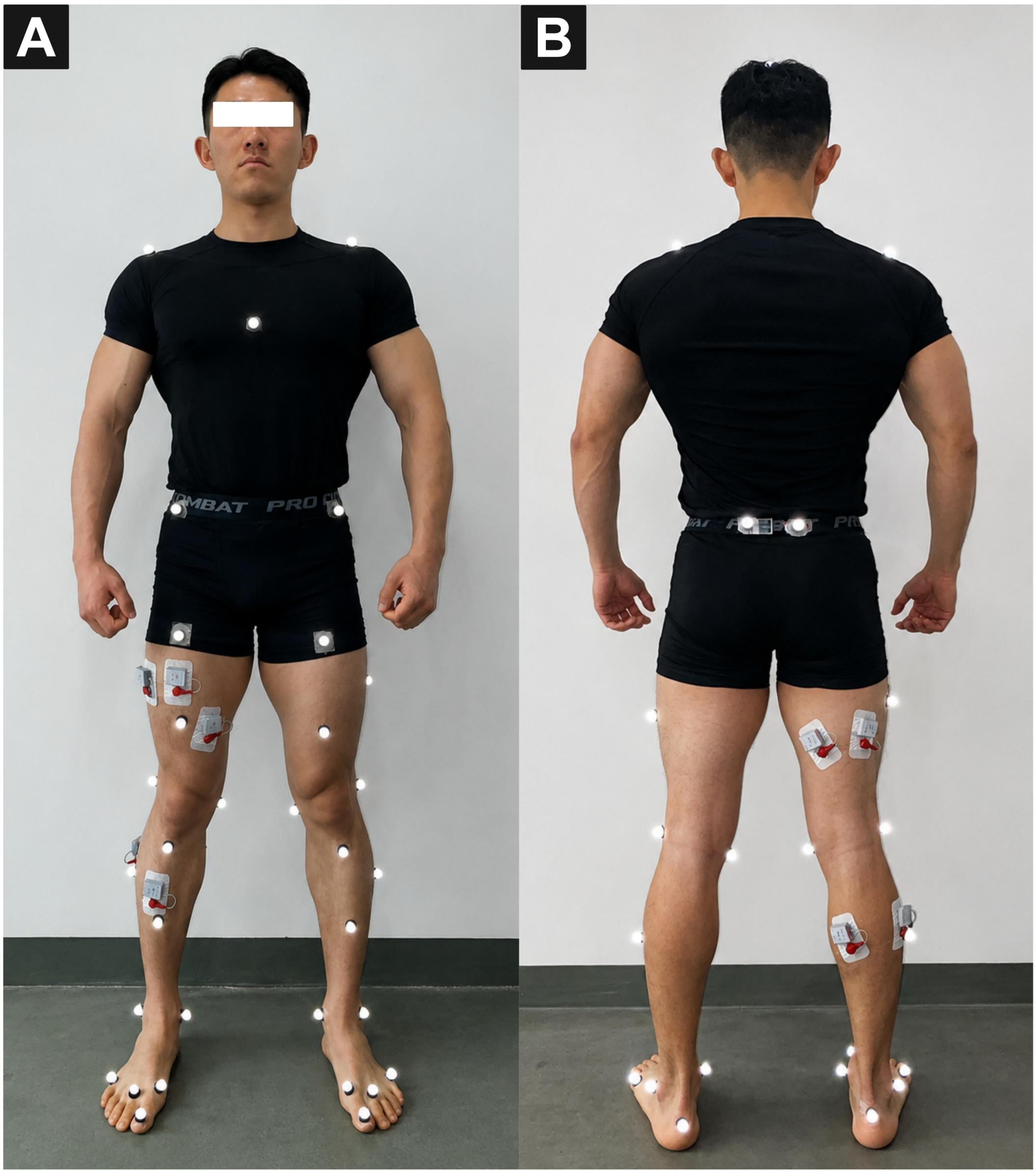

Supplementary Figure S1. Reflective marker configuration and surface electromyography (sEMG) electrode placement. Anterior (A) and posterior (B) views showing the reflective marker configuration and representative sEMG electrode placement used during the sit-to-walk task. Reflective markers were placed on major lower-limb anatomical landmarks according to the Vicon lower-limb marker set, and wireless bipolar sEMG electrodes were positioned over the target lower-limb muscles following SENIAM recommendations. The participant's eyes were masked to protect identity.

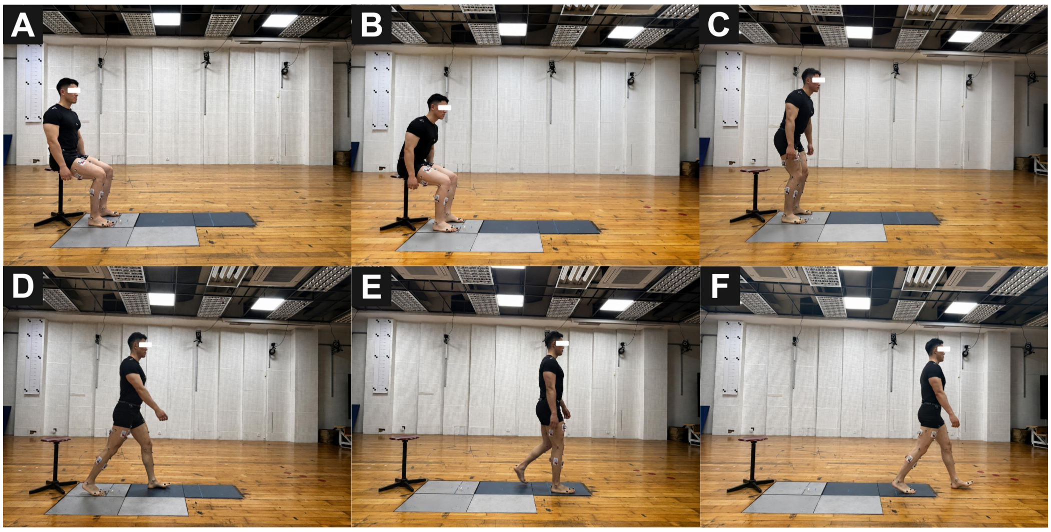

Supplementary Figure S2. Representative sequence of the sit-to-walk task.

Panels A–F show a representative sequence of the sit-to-walk task from quiet sitting to standing and subsequent gait initiation. Participants began from a standardized seated posture with both feet placed on the first force plate and were instructed to stand up and initiate forward walking after the start command. The participant's eyes were masked to protect identity.

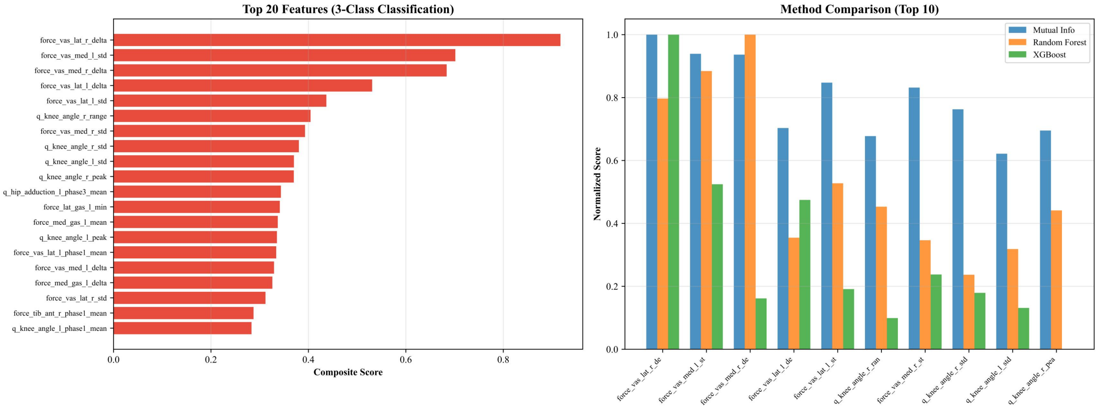

Supplementary Figure S3. Feature selection workflow and results. Composite importance scores were obtained by equally fusing normalized scores from mutual information, ANOVA F-tests, random forest, and XGBoost. Features selected by at least two methods were retained, yielding approximately 20 stable features for subsequent three-class modeling.

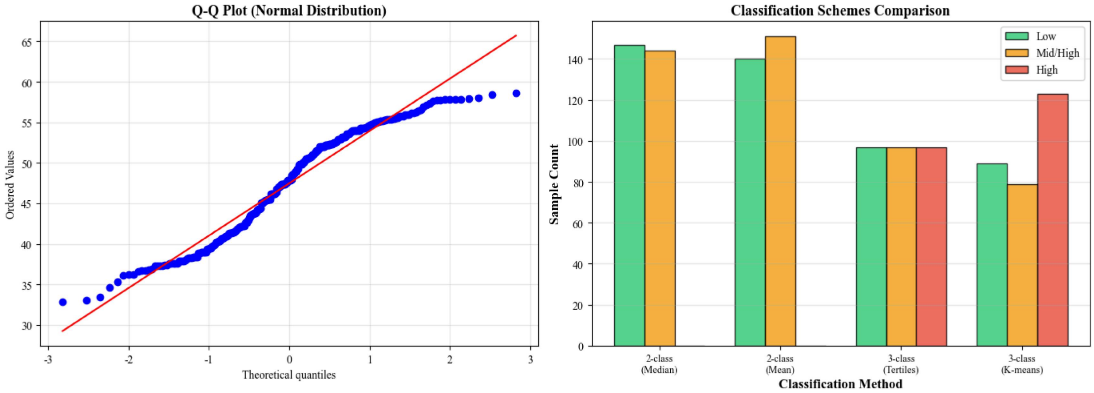

Supplementary Figure S4. Distribution of window-averaged metabolic power and tertile-based stratification into low, medium, and high groups, showing preserved gradient information and balanced class proportions (minimum class proportion >20%).

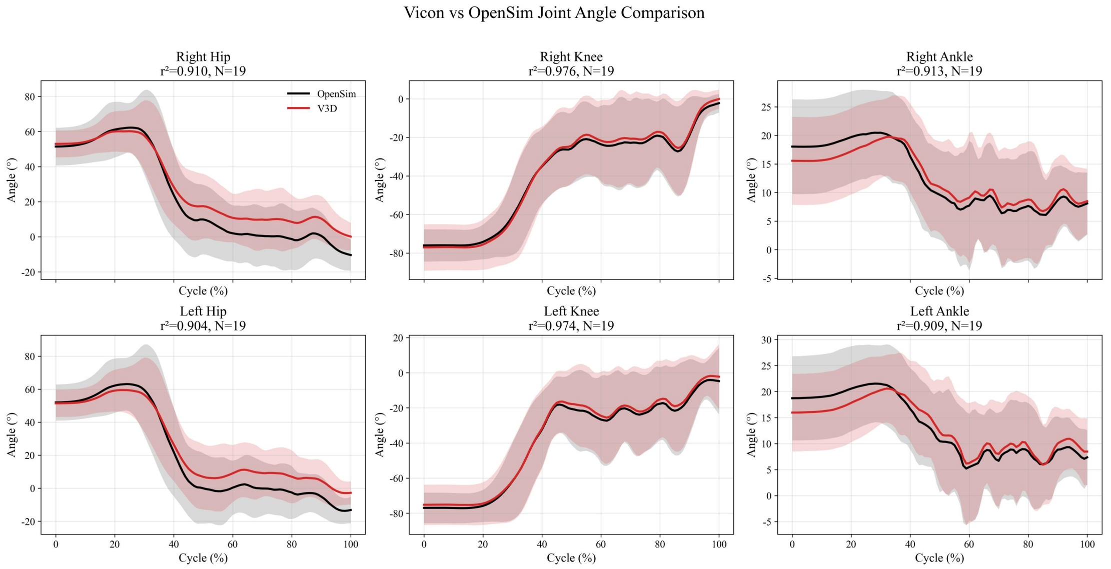

Supplemental Figure S5. Comparison of Vicon-based and OpenSim-derived lower-limb joint angles during the cycle.

Note. The right and left hip, knee, and ankle joint angle trajectories are shown as mean ± standard deviation across trials from 19 participants. Black curves represent joint angles derived from the OpenSim musculoskeletal model, whereas red curves represent Vicon-based joint angles.

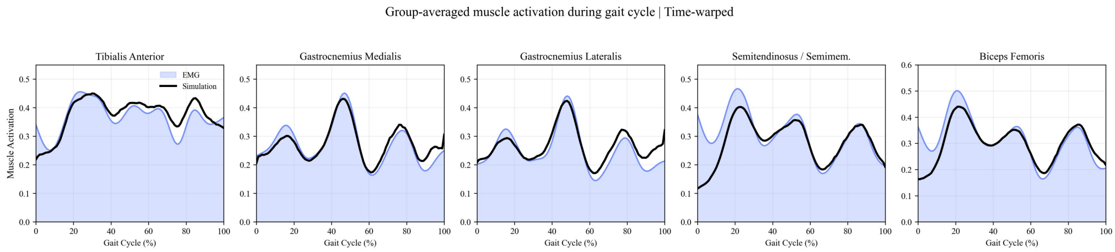

Supplementary Figure S6: Group-averaged comparison of EMG envelopes and OpenSim-simulated activations of representative lower-limb muscles during a complete gait cycle.

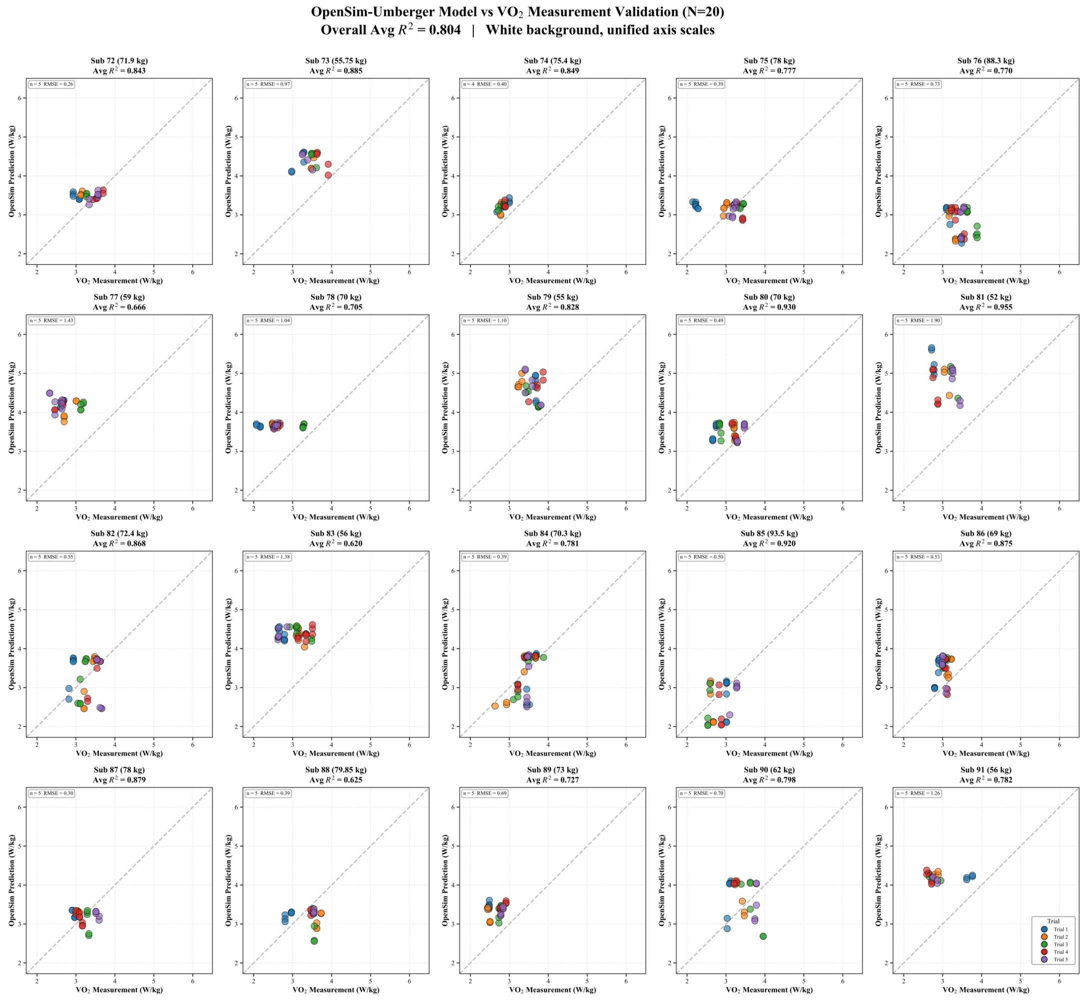

Supplemental Figure S7. Comparison of OpenSim–Umberger model-estimated metabolic rate and indirect calorimetry-measured oxygen consumption across five repeated STW trials.Trial labels (Trial 1–5) correspond to the original chronological order of the repeated STW trials performed during the experiment and were not reordered for analysis.

Supplementary Table S1: Best Hyperparameters for Each Model

Model	Best Hyperparameters
Random Forest	ccp_alpha: 0.01<br>class_weight: balanced<br>max_depth: 3<br>max_features: 4<br>max_leaf_nodes: 5<br>min_samples_leaf: 20<br>min_samples_split: 40<br>n_estimators: 20
Support Vector Machine	C: 0.5<br>class_weight: balanced<br>decision_function_shape: ovr<br>gamma: scale<br>kernel: rbf
XGBoost	colsample_bytree: 0.7<br>gamma: 0.1<br>learning_rate: 0.1<br>max_depth: 4<br>min_child_weight: 5<br>n_estimators: 50<br>reg_alpha: 0.5<br>reg_lambda: 1.0<br>subsample: 0.7
Logistic Regression	C: 0.1<br>class_weight: balanced<br>max_iter: 2000<br>multi_class: multinomial<br>penalty: l2<br>solver: saga
Gradient Boosting	learning_rate: 0.1<br>max_depth: 3<br>max_features: sqrt<br>min_samples_leaf: 10<br>min_samples_split: 20<br>n_estimators: 30<br>subsample: 0.7

K-Nearest Neighbors	metric: euclidean<br>n_neighbors: 15<br>p: 1<br>weights: distance
Multilayer Perceptron	activation: relu<br>alpha: 1.0<br>early_stopping: True<br>hidden_layer_sizes: (32, 16)<br>learning_rate: constant<br>learning_rate_init: 0.01<br>n_iter_no_change: 10<br>validation_fraction: 0.15
